# Two Cases of Aphasia

**Published:** 1875-08

**Authors:** Walter Hay

**Affiliations:** No. 163 State Street; Chicago


A MONTHLY RECORD OF

*Medicine, Surgery and the Collateral Sciences.*

EDITED BY J. ADAMS ALLEN, M.D., LL.D.: AND WALTER HAY, M.D.

VOL. XXXII. — AUGUST, 1875. — NO. 8.

### Original Communications.

ARTICLE I.

### TWO CASES OF APHASIA.

BY WALTER HAY, M.D.

CASE 1. *Traumatic Aphasia.* William Updegraf, æt. 40, farmer, came to Rush Medical College Clinic, Jan. 23rd, 1875, accompanied by a companion, who gave the following history of his case: Two years ago, while directing some repairs to his reaping machine, stooping to a horizontal position, watching the progress of a "bolt," which a smith was driving through a hole, he was struck upon the bridge of the nose by the end of the bolt suddenly driven through by an unusually powerful blow of the sledge. He was knocked down but recovered himself immediately, his nose bleeding copiously; soon becoming drowsy, he slept for about half an hour, when, awakening he rode three miles to his home and passed the afternoon and evening in attending to his ordinary duties, smoked a pipe after supper and retired to bed at his usual hour. His wife was awakened before daybreak by his stertorous breathing, and finding him entirely unconscious, sent for a physician. His condition was then one of complete unconsciousness—of which he has preserved no recollection subsequently; motility and sensibility upon the right side was completely lost; face drawn to the left; deglutition accomplished only when food was placed upon the root of the tongue; fæces and urine discharged involuntarily.

At the end of two weeks, consciousness returned, together with partial control over bladder and rectum—would indicate by signs his desire to evacuate these viscera. After six weeks was able to walk, dragging his right leg, with the assistance of a cane, but was unable to utter a sound. Up to the present time, his improvement has been gradual, and, for the most part, uniform. To-day he walks with a, scarcely perceptible, limp in the right leg, the toes being slightly inverted. No muscular atrophy—is able to pick up a pin from the palm of my hand with the thumb and forefinger of his right hand while looking at the object, but when his head is turned away cannot distinguish a fold of skin from the pin; grasps my hand firmly but with little force; very slight, scarcely perceptible, diminution of symmetry in the two sides of the face; cannot whistle, but can spit straight; has perfect control of muscles of eye-lids and eye-balls; tongue deflected to right (to paralyzed side); sensibility normal on both sides of tongue and face; sight, hearing, taste, smell, normal. His speech is limited to one word, "yes," which he articulates distinctly, but in a whisper. His intelligence appears to be perfect; exhibits much good humor and vivacity; enjoys a joke, laughs heartily, but inaudibly; answers questions quickly by an affirmative nod, or a negative shake of the head, as may be appropriate; writes his name William Updgr——, but shakes his head and cannot finish it—can write the initial letter of his residence S (Sycamore), but no more; readily distinguishes letters written in alphabetical order from those which are not. His general health is excellent, sleeps well, has a good appetite and digests his food well —bowels and kidneys act regularly.

Diagnosis—amnesic aphasia, from traumatic hæmorrhage.

Treatment—nutrition, diet, fresh air, absence of excitement, practice with the pencil daily and in the articulation of simple sounds.

CASE 2. *Aphasia from Embolism.* Rev. F—— P——, S. J., a prominent official of the Jesuit Society, consulted me, giving me the following history of his case. Is an Italian, 50 years of age, having the appearance of typical health—came from a long-lived race. Has had no illness except an attack of acute rheumatism when a lad in Italy; has passed thirty years in America, principally in the District of Columbia. His life has been one of severe study, and, during nine years—while Provincial of his Society—of great care. Eighteen months ago while celebrating mass at the altar of the church, found himself suddenly deprived of speech, to so great a degree that he was compelled to indicate his wishes to his servitors by signs. Had great difficulty in raising his right arm and hand. Retained sufficient self-control to finish his mass, although occupying about three times as long as usual. The loss of power in the right arm was also apparent in the leg, and was accompanied by numbness, pricking and tingling.

After the lapse of six months began to recover his faculty of speech, together with motion and sensation in the extremities, which had been only partially lost. He has now so far recovered, as to be able to walk with little perceptible difficulty; to write, although slowly and with care; to converse clearly upon any subject, but slowly and thoughtfully.

The most remarkable feature in the case, is the total obliteration from memory of certain items of acquired knowledge, previously in constant use; for example, he has forgotten entirely the "credo," being unable to recall a single word of it beyond the first. Murmur with regurgitation at the base of the heart.

Diagnosis—Embolism of left middle cerebral artery, from rheumatic endocarditis. Had been treated with K. Br. freely. Advised rest, mental and bodily, easy traveling and nutritious diet.

No. 163 State Street.

